# Animal product–free formation and cultivation of three-dimensional primary hepatocyte spheroids

**DOI:** 10.1016/j.dmd.2025.100147

**Published:** 2025-08-19

**Authors:** Evgeniya Mickols, Rejeen Mohammedamin, Lazaros Primpas, Stina Oredsson, Maria Karlgren

**Affiliations:** 1 Department of Pharmacy, Uppsala University, Uppsala, Sweden; 2 Department of Biology, Lund University, Lund, Sweden

**Keywords:** 3D PHH, Primary human hepatocytes, Fetal bovine serum, Serum-free, In vitro model

## Abstract

Three-dimensional (3D) cultures of primary human hepatocytes (3D PHH) are successfully used to reduce and replace the use of animal experiments in biomedical research. Yet, the initial formation of 3D PHH is highly dependent on the supplementation with FBS. However, the molecular composition of FBS and its effects on cultured cells are poorly understood. Moreover, FBS is prone to batch-to-batch variation, immunogenic risk, and lack of adherence to the replacement, refinement, and reduction of animal experiments. Here, we demonstrate that FBS can be fully replaced by animal-free substitutes, thus facilitating fully chemically defined and animal serum–free 3D PHH cultures. Specifically, we combined a previously developed animal-free substitute cocktail with a normoglycemic (5.5 mM glucose and 0.58 ng/mL insulin) chemically defined culture medium. Morphological and viability evaluations, along with global proteomics data, demonstrated that serum-free cultured 3D PHH have comparable viability and functional performance of cytochrome P450s, rendering this medium useful for long-term studies and in vitro absorption, distribution, metabolism, excretion, and toxicity applications. This study marks a significant advancement in the development of animal serum–free culture conditions for primary human cell cultures, paving the way for more reliable and ethical in vitro studies.

**Significance Statement:**

Most in vitro cell models rely on FBS. However, the use of FBS leads to inconsistent experimental results and raises serious ethical concerns. In this study, a chemically defined animal product–free cell culture medium with physiologically relevant levels of key hormones and nutrients for liver spheroid cultures was developed and evaluated. This study marks a significant advancement in the development of animal serum–free culture conditions for primary human cell cultures used in drug disposition studies.

## Graphical abstract

**Figure:**
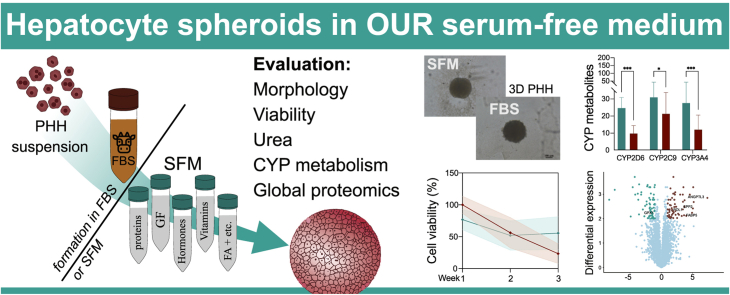

## 1 Introduction

In vitro primary human cell cultures are successfully applied to reduce and replace the use of animal experiments in biomedical research.^1^ Of note, relevant in vitro models even offer the potential to accelerate drug development and are increasingly incorporated into regulatory preclinical safety assessments.^2^ Although these in vitro models greatly contribute to the replacement, refinement, and reduction (3Rs) of animal experiments, culturing conditions for most in vitro models include the animal-derived component FBS.

The use of FBS is a routine cell culture practice. It has been used for the maintenance of cell cultures for nearly 70 years, and it remains the most common cell culture medium supplement.^3^ FBS is added to the culture medium to help the cells circumvent stress induced by the in vitro culture setting. However, the molecular composition of FBS is prone to batch-to-batch variation and is poorly understood at the molecular level.^4^, ^5^, ^6^ In addition, the use of FBS does not adhere to the 3Rs, as FBS production is associated with severe animal welfare issues.^7^

Although the problems associated with FBS are well-known, limited research has been dedicated to finding serum-free alternatives for in vitro models. Nonetheless, in recent studies focusing on serum replacement, the authors demonstrate that FBS-free media could be successfully developed and used to support in vitro cultures of various cell lines.^8^, ^9^, ^10^, ^11^, ^12^, ^13^, ^14^, ^15^ To substitute FBS, one would need to supplement cell culture medium with either human blood derivatives or a cocktail of human-derived or recombinant attachment, growth, and carrier proteins, as well as minerals, hormones, and vitamins. Such cell culture media are available as proprietary solutions from life science suppliers; however, the content of these media is undisclosed, which makes customization of the media or interpretation of the research results difficult. Alternatively, Rafnsdóttir et al^8^ recently published the composition of a fully defined animal-product–free and universal medium that could be used to effectively substitute FBS in long-term cultures in adherence to the 3Rs. As opposed to the proprietary cell culture media, the approach provided by Rafnsdóttir et al is fully disclosed and well-described, thus opening an opportunity for customization of serum-free medium (SFM) and adaptation of the basal components to the cell culture of choice. In addition, the paper by Rafnsdóttir et al has recently been supplemented with 2 detailed methodological papers covering all aspects of the animal serum–substitute preparations and use,^15^^,^^16^ along with a comprehensive cost estimate to facilitate easy price comparisons.^16^

In this paper, we focus on developing and evaluating FBS-free 3D primary human hepatocyte (3D PHH) cultures for drug disposition studies. 3D PHH has recently emerged as a gold standard model for the prediction of liver toxicity and drug clearance.^17^, ^18^, ^19^, ^20^, ^21^, ^22^ Notably, from a 3Rs perspective, 3D PHH are nearly an ideal in vitro model; it is sourced from surplus human tissue and cultured in a high-throughput microwell format in a chemically defined medium with minimal supplementation. Yet, the initial formation of 3D PHH has so far been highly dependent on the supplementation of the medium with FBS. This decreases the 3Rs compliance of 3D PHH, and possibly, tampers with the desired absorption, distribution, metabolism, excretion, and toxicity (ADMET)–related properties due to FBS batch-to-batch variability. Thus, FBS-free 3D PHH formation is desirable. Of note, we previously demonstrated that 3D PHH can be cultured in chemically defined, physiologically relevant culture medium with fasting levels of glucose and insulin for at least 2 to 3 weeks.^17^^,^^20^ Importantly, this condition supports the hepatic phenotype and the expression of ADMET-related proteins and their functions.

Here, we transition the 3D PHH system to a fully animal serum–free physiologically relevant model and comprehensively benchmark its performance and utility for preclinical pharmacological applications. Specifically, we compared 3D PHH spheroid formation, viability, urea production, cytochrome P450 (P450) activity, and the global proteome of spheroids formed in an FBS-supplemented medium with those formed in the fully defined serum-free condition. We demonstrate that chemically defined animal serum–free culture medium could replace FBS-supplemented medium and potentially improve 3D PHH culture stability, long-term P450 metabolic function, and reproducibility.

## 2 Materials and methods

### 2.1 Isolation of primary human hepatocytes

Human hepatocytes were isolated from histologically normal surplus liver tissue obtained from cancer patients undergoing liver resections at the Department of Surgery at Uppsala University Hospital. Donors signed informed consent in agreement with the approval from the Uppsala Regional Ethical Review Board (Ethical Approval no. 2009/028, amended 2018/1108). A previously described 2-step perfusion procedure for hepatocyte isolation was performed.^23^ Briefly, the liver tissue was rinsed of excessive blood with Hypothermosol FRS (Biolife Solutions) and perfused with collagenase and protease buffers for tissue digestion. Isolated hepatocytes were further centrifuged with isotonic Percoll for debris removal and cryopreserved until further use as previously described.^24^, ^25^, ^26^

### 2.2 3D Primary human hepatocyte spheroid culture

**Fig. 1:**
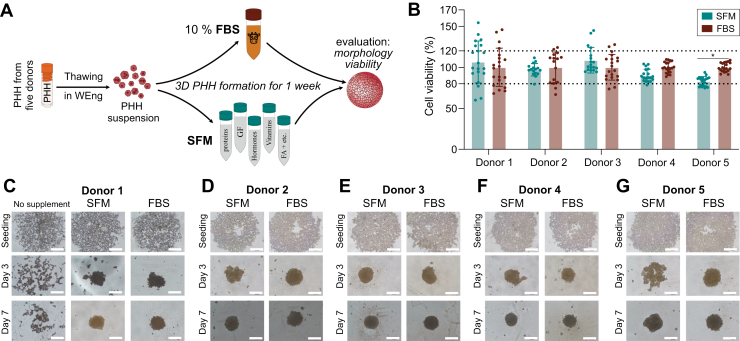
3D primary human hepatocyte spheroid (3D PHH) formation in serum-free medium (SFM) or conventional FBS-supplemented medium. (A) Schematic overview of the 3D PHH seeding and culture maintenance procedures for short-term experiments. PHH were thawed in William’s E normoglycemic medium (WEng) and seeded with 10% FBS or serum substitute according to Rafnsdottir et al.^8^ After full spheroid formation (1 week), the culture medium was exchanged for unsupplemented WEng. (B) Viability (ATP content) of 3D PHH from 5 different donors in SFM or with FBS supplementation 1 week after seeding. Data are presented as mean ± SD for viability measurements normalized to conventional conditions with FBS supplementation (*n* = 16–20; see Supplemental Table 7 for detailed information). The dotted lines indicate viability ratios of 80% to 120%. ∗*P* < .005; in two-way ANOVA with Šídák’s multiple comparisons test. (C–G). 3D PHH formation in 5 donors. Bright-field microscopy images were taken at seeding, after the third day in culture, and after 1 week in culture. Scale bar, 250 *μ*m.

Five PHH donors were used in this study (Supplemental Table 1). Cryopreserved hepatocytes were gently thawed and transferred to isotonic 27% Percoll (GE Healthcare) in Williams E normoglycemic medium (PAN-Biotech GmbH) without serum (from here and further referred to as WEng; full medium composition is provided in Supplemental Table 2) and centrifuged at 100 × *g* for 10 minutes.^17^^,^^20^ After the centrifugation, the supernatant with cell debris and dead cells in Percoll was discarded. The hepatocytes were resuspended in a small volume of warm WEng medium, and cell viability and count were measured with acridine orange−propidium iodide staining using a Cellometer Vision CBA image cytometer (Nexcelom Bioscience). Then, hepatocytes were resuspended either in WEng medium supplemented with 10% FBS or with the serum substitute, containing, for example, recombinant hormones, proteins, growth factors, etc, developed by Rafnsdóttir et al.^8^ The full composition of the serum substitute is provided in Supplemental Table 2. The PHH suspensions were seeded at 2000 cells/well in 100 *μ*L of cell culture medium in ultralow attachment 96-well plates Corning 7007 (Corning), sedimented by 100 × *g* centrifugation for 5 minutes, and incubated at 37 °C in a humidified incubator with 5% CO_2_ in air (Fig. 1A). The first medium change was typically performed after the initial spheroid formation (5–7 days after the seeding of PHHs), starting with a 50% medium change to WEng without any additional serum/serum-substitute supplement. Spheroids were maintained in unsupplemented WEng for up to 3 weeks of overall culture time, with a medium change every 48 to 72 hours. All media and cell-culture supplements were purchased from VWR, Thermo Fisher Scientific, or Sigma-Aldrich unless otherwise stated. The serum substitute cocktail developed by Rafnsdóttir et al was prepared according to Weber et al in Oredsson’s laboratory, Lund University, and the composition of the supplement is provided in Supplemental Table 2.^8^^,^^15^

### 2.3 PHH viability assay

PHH viability/health state was assessed using the CellTiter-Glo 3D assay (Promega) according to the manufacturer’s instructions. Briefly, the 3D PHH plates and CellTiter-Glo 3D reagent were equilibrated to room temperature for approximately 30 minutes, and the CellTiter-Glo 3D reagent was added to the well in a volume equal to the cell culture medium. The plates were incubated on a PST-60HL-4 Plate shaker-thermostat for approximately 30 minutes to facilitate the disintegration of the spheroids. The luminescence was then measured in a TECAN Spark plate reader for a minimum number of 15 spheroids per donor and condition. Data were normalized to results for 3D PHH formed in FBS at week 1.

### 2.4 Urea production assay

Urea production was assessed using the Urea Nitrogen Colorimetric Detection Kit (Thermo Fisher Scientific) according to the manufacturer’s instructions. Briefly, culture medium was collected from 3D PHH at weeks 1, 2, and 3 and stored frozen until analysis. Before analysis, the medium was thawed, and all reagents were equilibrated to room temperature. To 50 *μ*L of medium, 75 *μ*L of each reagent was added, followed by incubation at room temperature for 30 minutes. Absorbance was then measured at 450 nm using a TECAN Spark plate reader and quantified against the standard curve provided in the kit. For each condition and donor, 4 biological replicates were analyzed, each consisting of medium pooled from 6 spheroids.

### 2.5 Cytochrome P450 activity

Single 3D PHH were incubated for 8 hours at 37 °C in WEng medium with a cocktail of P450 substrates: 10 *μ*M midazolam (CYP3A4), 10 *μ*M diclofenac (CYP2C9), 10 *μ*M bufuralol (CYP2D9), and 10 *μ*M bupropion (CYP2B6; donor 1 only). After 8 hours, the reactions were stopped by snap-freezing the samples. For analysis, samples were thawed and diluted with acetonitrile (sample:acetonitrile 60:40) supplemented with 10 nM warfarin as an internal standard. Samples were centrifuged twice for 10 minutes at 4 °C with 3500 × *g* to sediment cell pellets and proteins, and concentrations of the respective P450 metabolites (1-hydroxymidazolam, 4-hydroxydiclofenac, 1-hydroxybufuralol, and hydroxybupropion) in supernatants were analyzed on a Waters Acquity UPLC coupled to Waters Xevo TQ MS with electrospray ionization (liquid chromatography conditions and tandem mass spectrometry (MS/MS) transitions are provided in Supplemental Tables 3 and 4, respectively). Compounds were separated with a 2-minute gradient elution of acetonitrile and 0.1% formic acid (flow rate 0.5 mL/min) on a Waters BEH C18 column, 2.1 × 50 mm (1.7 *μ*m) at 60 °C. Peaks were quantified using MassLynx Software V4.2 with the TargetLynx module using the internal standard and calibration curves.

### 2.6 Global proteomics analysis

#### 2.6.1 Sample preparation procedure

3D PHH (at minimum one 96-well plate of spheroids per condition and 4 biological replicates) were collected in low protein binding microcentrifuge tubes (Eppendorf, Fisher Scientific), washed twice with ice-cold PBS, and snap-frozen in liquid nitrogen. Then samples were rapidly defrosted, and proteins were prepared using the iST sample preparation kit (PreOmics) according to the instruction manual with the exception of the reconstitution buffer that was exchanged for 0.1% formic acid in hypergrade water. Final peptide concentrations were determined by fluorometric quantification with the Protein Broad Range Assay on the Qubit 4 Fluorometer (Thermo Fisher Scientific).

#### 2.6.2 Liquid chromatography-tandem mass spectrometry analysis of the peptides

Peptides were separated on an EASY-spray C18-column (50 cm, 75 *μ*m inner diameter), using an acetonitrile/water gradient (0.1% formic acid) at 300 nL/min. Eluted peptides were analyzed using the TopN method (full MS followed by ddMS2 scans) on an Orbitrap Q Exactive HF mass spectrometer (Thermo Fisher Scientific), operating in a data-dependent mode with survey scans at a resolution of 120,000, automatic gain control target of 3 × 10^6^, and maximum injection time of 120 ms. The top 15 most abundant isotope patterns were selected from the survey scan with an isolation window of 1.7 m/z and fragmented with normalized collision energy at 26. The MS/MS analysis was performed with a resolution of 30,000, an automatic gain control target of 1 × 10^5^, and a maximum injection time of 50 ms. Blanks were injected between every sample to ensure minimal transfer of peptides between biological replicates and different conditions. The mass spectrometry proteomics data have been deposited to the ProteomeXchange Consortium via the PRoteomics IDEntifications (PRIDE) partner repository with the data set identifier PXD056428.^27^^,^^28^

#### 2.6.3 Data analysis

The raw MS datafiles were processed using MaxQuant version 2.5.2.0.^29^ Proteins were identified by searching MS and MS/MS data of peptides against a reference human proteome database retrieved from the UniProtKB/Swiss-Prot curated database on 2024-05-11 and contaminant FASTA provided by MaxQuant.^30^ A description of the parameters used for peptide identification by MaxQuant can be found in the mqpar.xml uploaded to PRIDE/PXD056428. Briefly, carbamidomethylation was set as a fixed modification, whereas oxidation and acetylation were variable modifications; the match between runs algorithm was used; decoy sequences were created by reversing the target sequences, and peptide-spectrum matches, peptides, and proteins were validated at a 1% false discovery rate estimated using the decoy hit distribution. Quality control of the MaxQuant search was performed using an R-based quality control pipeline called Proteomics Quality Control version v1.0.16.^31^

Subsequent data cleanup was performed using an in-house developed proteomics data pipeline in development, R version 4.3.0. Concisely, the ProteinGroups data table was cleaned up using the Tidyverse package, and raw intensities were normalized using variance stabilization normalization using the variance stabilization normalization package (Bioconductor 3.17 release).^32^^,^^33^ Protein abundances (fmol/*μ*g total protein) were calculated with the total protein approach.^34^ Data overview analysis was performed in Perseus version 2.0.10.0.^35^ Differential expression (DE) analysis was implemented via the Amica web platform version 3.0.1.^36^ Gene ontology enrichment analysis was performed using the GOrilla tool.^37^^,^^38^ Normalized intensities table: protein abundances generated using total protein approach and results of DE are summarized in Supplemental material.

### 2.7 Quantification and statistical analysis

Unless otherwise stated, statistical analysis and plot generation were carried out using GraphPad Prism version 10 (GraphPad Software). All results are presented as mean values ± SDs of at least 3 biological replicates if not otherwise specified.

## 3 Results

### 3.1 Spheroid formation in animal serum-free medium

To be used in an experimental setting, PHH need to be successfully assembled into spheroids. Because extracellular matrix and transmembrane cell adhesion proteins are typically lost due to the digestion associated with the PHH isolation procedure,^17^ it is nearly impossible for 3D PHH to self-assemble without additional structural proteins with preserved phenotype in a reasonable experimental time (Supplemental Fig. 1). To circumvent this limitation and combat additional cell stress associated with cryopreservation, PHH are typically thawed and seeded in a medium supplemented with FBS.^17^^,^^18^^,^^20^

Here, we used a humanized serum-substitute cocktail developed by Rafnsdóttir et al^8^ to substitute FBS during the spheroid formation time (Fig. 1A). Intriguingly, PHH efficiently assembled into well-defined viable 3D spheroids in the SFM by the seventh day in culture (Fig. 1, B–G). We observed this successful 3D PHH formation in SFM across 5 different donors, yet the speed of this process and morphology of spheroids in both SFM and FBS varied (Fig. 1, C–G). Thus, we asked five 3D PHH experts, who are not involved in the current study, to blindly assess the morphology (in terms of compactness, rim clarity, and presence of debris) of the 3D PHHs formed in SFM or FBS after 1 week of culture time (Supplemental Tables 5 and 6). As expected, 3D PHH from different donors were scored differently (average score 2.9–4.1). However, 3D PHH formed in SFM obtained higher or similar scores compared with the scores for 3D PHH formed in FBS, further confirming the successful microtissue formation in FBS-free conditions.

Additionally, to assess the formed 3D PHH from 5 biological donors, we performed ATP content measurement as an estimation of viability 1 week after the seeding (Fig. 1B). For all 5 PHH donors, the viability of the 3D PHH formed in SFM stayed within control levels, that is, within 80% to 120% change normalized to the conventional FBS setting. For donor 5, a statistically significant reduction in viability was seen for the 3D PHH formed in SFM (Fig. 1B). Nonetheless, the viability was still within the acceptance limit (82% of the control values).

### 3.2 Long-term cultures

**Fig. 2:**
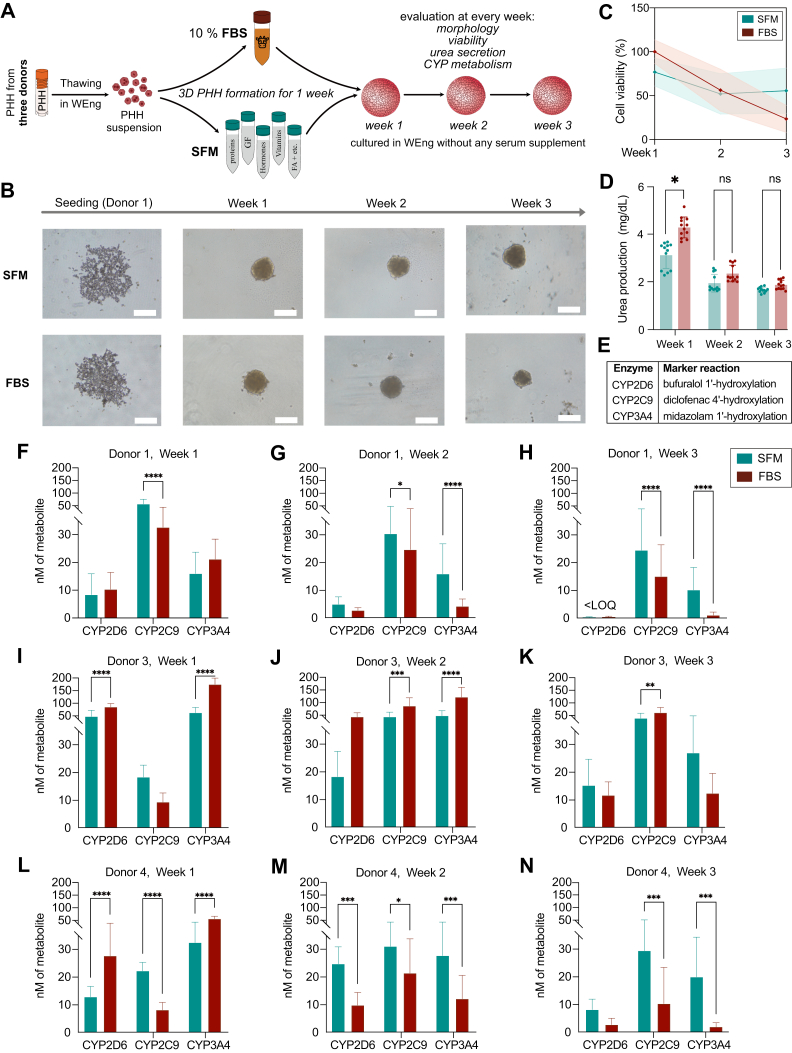
3D primary human hepatocyte spheroid (3D PHH) formation and function for 3 weeks. (A) Schematic overview of the 3D PHH seeding and culture maintenance procedures for long-term experiments. PHH were thawed in William’s E normoglycemic medium (WEng) and seeded with 10% FBS or serum substitute according to Rafnsdottir et al.^8^ After full spheroid formation (1 week), the culture medium was exchanged to unsupplemented WEng, and morphology, viability, urea production, and cytochrome P450 (P450) metabolism were evaluated at weeks 1, 2, and 3. (B) Donor 1 3D PHH spheroid formation in serum-free medium (SFM) or FBS-supplemented medium and corresponding morphology for 3 weeks in culture. Scale bar, 250 *μ*m. (C) Mean viability (ATP content) for 3D PHH of donors 1, 3, and 4 for 3 weeks in culture in SFM (green) or FBS-supplemented medium (brown); the shaded area represents SD (*n* = 15–24, see Supplemental Table 8 for detailed information). (D) Urea production in 3D PHH of donors 1, 3, and 4 for 3 weeks in culture in SFM (green) or FBS-supplemented medium (brown). Data are presented as mean ± SD (*n* = 4, where each replicate consists of medium pooled from 6 spheroids). ∗*P* < .0001; ns, not significant using two-way ANOVA with Šídák’s multiple comparisons test. (E) Marker reactions used for assessing P450 function. (F–N) Marker metabolite formation in nM in spheroids cultured for 1, 2, and 3 weeks. Data are presented as mean ± SD (*n* = 11–40; see Supplemental Table 9 for detailed information). ∗∗*P* < .01; ∗∗∗∗*P* < .0001; ns, not significant using two-way ANOVA with Šídák’s multiple comparisons test. GF, growth factors; FA, fatty acids; <LOQ, below the limit of quantification.

As 3D PHH are often used for long-term cultures, 3 of the 5 PHH donors (donor 1, 3, and 4) were seeded in either SFM or FBS-supplemented medium, and these 3D PHH cultures were used to verify that animal serum–free conditions were suitable for long-term studies (Fig. 2A). In addition, a global proteomics analysis was conducted over a 3-week culture period for 3D PHH from donor 1.

#### 3.2.1 Spheroid morphology

For long-term studies, we performed further evaluations of the morphology of 3D PHH from donors 1, 3, and 4 through 3 weeks in culture—a typical timeline for experiments involving this in vitro model (Fig. 2B and Supplemental Fig. 2).^17^^,^^20^^,^^22^^,^^39^^,^^40^ We observed that the successfully formed spheroids preserved their morphology and integrity for the whole duration of the experiment (3 weeks), independently of whether the 3D PHH were formed with the support of FBS or in SFM.

#### 3.2.2 Viability of long-term cultures

Likewise, we measured viability in 3D PHH from donors 1, 3, and 4 over the culture period of 3 weeks (Fig. 2C, Supplemental Fig. 3). Interestingly, although the average normalized ATP levels in 3D PHH formed under SFM conditions were slightly lower than those in FBS-formed 3D PHH during the first week of culture, a notable decrease in ATP quantities (1.8-fold) was seen for spheroids formed in FBS by the second week in culture, with viability continuing to decrease during the third week. Meanwhile, ATP levels in 3D PHH formed in SFM decreased only by 1.5-fold by the second week and then remained stable for the remainder of the culture period.

#### 3.2.3 Urea production in long-term cultures

Additionally, urea production was evaluated in 3D PHH from donors 1, 3, and 4 over the 3-week culture period (Fig. 2D and Supplemental Fig. 4). At week 1, spheroids formed in FBS showed significantly higher urea levels compared with those formed in SFM. However, by weeks 2 and 3, no statistically significant differences were observed between the 2 conditions.

#### 3.2.4 Cytochrome P450 activity

3D PHH are increasingly used in drug disposition and metabolism studies.^19^^,^^21^^,^^22^^,^^41^^,^^42^ To validate the applicability of 3D PHH formed in SFM for such studies, we measured the activities of common drug-metabolizing P450 enzymes at 1, 2, and 3 weeks postseeding (Fig. 2, E–N; data normalized to average viability available in Supplemental Fig. 5). As expected, variability was observed both between donors and between weeks in culture. Overall, 3D PHH formed in SFM performed equally well or better than those formed in FBS at later time points, whereas FBS-formed 3D PHH tended to perform better during the early stages of culture. Specifically, for donors 1 and 4, we observed higher CYP2C9, CYP2D6, and CYP3A4 activity in SFM cultures compared with FBS at various time points.

#### 3.2.5 Global protein expression

**Fig. 3:**
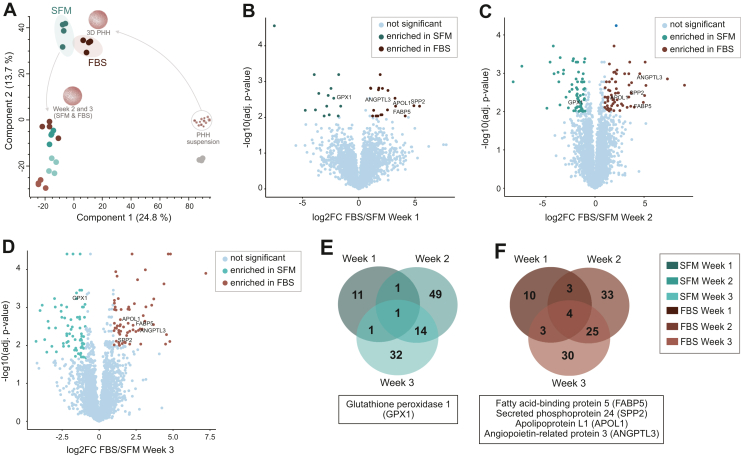
Proteomes of primary human hepatocyte spheroids (3D PHH) from donor 1 formed in serum-free medium (SFM) or FBS-supplemented medium. (A) Principal component analysis of the global proteomes for the 3 culture weeks based on all identified proteins (4 biological replicates per condition each consisting of spheroids pooled from one 96-well plate, 4506 identified proteins). The number in parentheses is the percentage of variability explained by each component. (B–D) Differential expression analysis for 3D PHH cultures at weeks 1, 2, and 3, respectively. Log2 fold change-threshold = 1; significance threshold for multiple-hypothesis adjusted *P* value = .01. (E, F) Venn diagrams of the enriched differentially expressed proteins between 3D PHH formed with SFM or with FBS for 1, 2, and 3 weeks. Consistently enriched differentially expressed proteins for SFM and FBS (panels E and F, respectively) are highlighted in the boxes underneath the Venn diagrams and in panels B, C, D (proteomes correspond to donor 1 cultures shown in Fig. 2B.)

Next, we investigated whether the use of SFM during the 3D PHH formation affected the proteomes of 3D PHH for donor 1. In total, 4506 proteins were identified, and to obtain a global view of the data set, we applied principal component analysis (PCA). PCA indicated a clear, and expected, separation between proteomes of uncultured PHH in suspension and in 3D spheroid format, with the first component explaining 24.8% variance (cf.^17^; Fig. 3A). Interestingly, along the second component (13% variance), the data separated mostly based on the culture time, and the difference between proteomes of 3D PHH formed in the 2 different conditions was not a driving factor of the separation (Fig. 3A). The PCA data indicated that the shift from the conventional FBS-based 3D PHH protocol to the SFM protocol did not have a major impact on the proteomics fingerprint of 3D PHH in vitro cultures. However, we still observed that 3D PHH formed in these 2 conditions formed subclusters on the PCA plot.

To further investigate possible differences between the conditions, we performed DE analysis (Fig. 3, B–D and Supplemental material). During the first week of 3D PHH cultures, we detected 34 differentially expressed proteins (DeqMS, Log2 fold change threshold = 1, significance threshold for multiple-hypothesis adjusted *P* value = .01). Unexpectedly, we detected more differentially expressed proteins by the second week postseeding—130 proteins at the same statistical cut-off (Fig. 3C and Supplemental material DE W2). We found no enriched pathways in 3D PHH formed in SFM but observed multiple biological processes to be significantly altered in 3D PHH formed in FBS-supplemented medium (Supplemental material DE W2), for example, negative regulation of catalytic activity (*q* = 9.42E-5, *e* = 4.89) and negative regulation of molecular function (*q* = 1.62E-3, *e* = 3.79). Likewise, after 3 weeks in culture, we found 110 proteins to be differentially expressed (Fig. 3D) and observed significant changes only in the 3D PHH formed in FBS-supplemented medium, which again exhibited negative changes in catalytic activity and other similar metabolic changes (Supplemental material DE W3). Nonetheless, almost no differentially expressed proteins across all conditions were connected with meaningful alterations in specific hepatic functions. Furthermore, we observed only a minor portion of the proteomes to be differentially expressed (0.8%, 2.9%, and 2.4% for weeks 1, 2, and 3, respectively; Fig. 3, B–D). Also, out of all differentially expressed proteins, only glutathione peroxidase 1 (GPX1) was consistently upregulated in spheroids formed in SFM during the whole duration of culture time (Fig. 3E), whereas in 3D PHH formed in FBS-substituted medium, fatty acid-binding protein 5, secreted phosphoprotein 24, apolipoprotein L1, and angiopoietin-like 3 were consistently upregulated (Fig. 3F) when compared with SFM.

#### 3.2.6 Expression of clinically relevant ADMET proteins

**Fig. 4:**
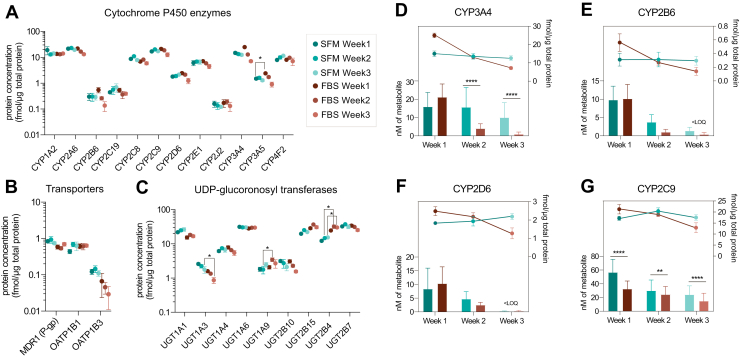
Global proteomics profiling in donor 1 of the absorption, distribution, metabolism, excretion, and toxicity-related proteins highlighted in ICH M12 guidelines. (A) Expression levels of quantified cytochrome P450 (P450) drug-metabolizing enzymes. (B) Expression levels of quantified uridine diphosphate (UDP)-glucuronosyl transferases (UGTs). (C) Expression levels of quantified drug transporters. Datapoints in all graphs represent mean expression values of 4 biological replicates with SD. Stars signify differentially expressed proteins in differential expression analysis. (D–G) Side-by-side comparison of expression and activity of CYP3A4, CYP2B6, CYP2D6, and CYP2C9, respectively. <LOQ, below the limit of quantification.

To further support the applicability of 3D PHH formed in SFM in drug development studies, we analyzed the expression levels of the clinically relevant ADMET proteins mentioned in the recently released International Council for Harmonisation of Technical Requirements for Pharmaceuticals for Human Use (ICH) M12 guidelines on drug interaction studies (Fig. 4).^43^ Overall, the expression of ADMET proteins across all conditions was stable. We found no statistical difference in P450 expression levels, except for lower expression of CYP3A5 in spheroids formed in SFM during the first week of in vitro culture (Fig. 4A). Likewise, we did not detect any significant expression differences for the P-glycoprotein (P-gp/MDR1) and organic anion transporting polypeptide (OATP) 1B1 and 1B3 transporters (Fig. 4B).

Interestingly, among the ADMET proteins, we found the most statistically significant differences in uridine 5’-diphosphate-glucuronosyltransferases (UGTs) expression, with UGT1A3 being upregulated in 3D PHH formed in SFM during the third week of culture, while UGT1A9 and UGT2B4 were downregulated in the same condition during the second or second and third weeks of culture, respectively (Fig. 4C).

Lastly, we compared the expression levels and the obtained activity of P450s (Fig. 4, D–G). Similarly to what has been demonstrated by others, we observed that in conventional FBS-based conditions, P450 activity and expression decreased in 3D PHH along the culture time (cf.^17^^,^^19^; Figs. 2, F–H and 4, D–G). However, we noted that for this donor, 3D PHH formed in SFM preserved a stable expression of P450s over 3 weeks of culturing. In addition, we observed P450 activity to be notably more stable in 3D PHH formed in SFM, in particular for CYP2C9 and CYP3A4 (Figs. 2, F–H and 4, D–G).

## 4 Discussion

Although earlier studies have explored the possibility of ADMET models being introduced at least temporarily to serum-free conditions,^17^, ^18^, ^19^, ^20^^,^^44^, ^45^, ^46^, ^47^ fully chemically defined 3D PHH cultures, from the PHH seeding until the culture termination, have not been evaluated. Here, we used the serum-free cocktail developed by Rafnsdóttir et al,^8^ and followed up on the series of papers from our laboratory that demonstrate excellent PHH performance in a physiologically relevant culture medium supplemented with fasting levels of insulin and glucose.^17^^,^^20^

In this context, besides being animal-derived and thus inconsistent with 3Rs principles, FBS also poses practical challenges, particularly its well known batch-to-batch variability, which can influence downstream assays.^6^ This is well established for cell lines, where many laboratories test several FBS batches in order to identify the most suitable one before purchasing in bulk. In our laboratory, we have observed such variability in growth, morphology, monolayer integrity, and transporter function in colon adenocarcinoma (Caco-2), Madin Darby canine kidney, and stably transfected human embryonic kidney (HEK293) cells (Karlgren, unpublished results). To our knowledge, FBS batch-to-batch effects have not been systematically studied in PHH or during 3D PHH formation. Still, studies on other cell types have shown that FBS concentration can influence the formation of MCF-7 spheroids,^48^ and that the type of serum used (human or bovine) can affect spheroid formation in SiHa and HeLa cells.^49^ Although results from immortalized cell lines cannot be directly extrapolated to primary cells, they support the possibility that similar variability may affect 3D PHH cultures. Notably, to further enhance reproducibility, the defined SFM could be refined by substituting human-derived proteins (eg, plasma-derived albumin or placenta-derived laminins) with recombinant counterparts. This refinement would help eliminate batch-to-batch variability while also supporting the development of fully defined, animal-free models in accordance with the 3Rs objectives.

Building on this, in all standard 3D PHH culture protocols, FBS is used at seeding.^17^^,^^18^^,^^20^^,^^50^, ^51^, ^52^ This is because adhesion proteins and supplements present in FBS appear to be necessary for spheroid formation. Thus, an addition of FBS (or serum substitute) seems crucial for PHH self-assembly. Also, here we show that PHH seeded completely without any supplements do not easily or just never form spheroids (Fig. 1C, Supplemental Fig. 1). This could be explained by the natural synthesis of structural proteins by the liver, which the hepatocytes are responsible for in the body. In contrast, PHH seeded in a medium supplemented with the serum-free cocktail suggested by Rafnsdóttir et al*,*^8^ successfully formed spheroids within 1 week of culture time, just like the PHH seeded in FBS-containing medium using the standard protocol. Not only did spheroids form in SFM conditions, but after 1 week in culture their morphology, assessed in terms of compactness, rim clarity, and presence of debris, was comparable to or exceeded that of spheroids formed from PHH from the same donor under FBS-supplemented conditions, as evaluated by blinded researchers familiar with 3D PHH studies (Supplemental Tables 5 and 6).

Naturally, we observed an interdonor variability both in the time needed for spheroid formation and in morphology. Yet all these variations were expected and within biological limits, as PHH donor batch variability is a well known phenomenon.^24^, ^25^, ^26^ This is once again reflected in the morphological score granted by our fellow researchers and our viability measurements.

Further, we continued to track the viability, urea production, and morphology of 3D PHH from 3 selected donors (donors 1, 3, and 4) for 3 weeks. We observed that spheroids formed in either SFM or FBS retained their morphology for all 3 weeks (Fig. 2B, Supplemental Fig. 2); however, in line with previous observations reported in the literature, FBS-formed spheroids from one of the donors (donor 1) showed a certain degree of compactness by the second and third week in culture (Fig. 2B, Supplemental Fig. 6).^18^^,^^52^ Additionally, and consistent with previous reports, a decrease in overall ATP content/viability was observed in spheroids formed in FBS between the first and second weeks of culture.^17^^,^^19^^,^^53^ Although the initial ATP levels were lower in 3D PHH formed in SFM, the decline over time was less marked as compared with FBS-formed spheroids (Fig. 2C). Instead, ATP levels remained relatively stable throughout the observation time, especially during weeks 2 to 3. Similarly, urea production was initially lower in 3D PHH formed in SFM at week 1, but no significant differences were observed between the 2 conditions at weeks 2 and 3 (Fig. 2D).

Interestingly, the P450 data and proteomics data for donor 1 support our observations regarding viability and morphology. Overall, P450 protein expression levels remained more stable in 3D PHH formed in SFM, with the activity of CYP3A4 and CYP2C9 being significantly higher in 3D PHH formed in SFM, particularly during the second and the third weeks of culture. A decrease in P450 activity in spheroids formed in FBS could be connected to a decrease in ATP content. Kanebratt et al^19^ have previously demonstrated that P450 activity follows along with ATP content, and when the P450 metabolites formation rate is normalized to ATP levels—metabolic formation comes across as stable. Indeed, normalization to average viability resulted in more comparable metabolic activity profiles over time (Supplemental Fig. 5); nevertheless, metabolite formation remained higher in the SFM condition at later time points, albeit to a lesser extent.

Considering that only minor differences were seen in morphology and functional performance of 3D PHH formed in FBS-supplemented medium or SFM, we did not expect the proteome of donor 1 to show any major differences between the 2 conditions. Indeed, global proteomics analysis revealed a high degree of similarity in the proteomics fingerprint of these cultures. In DE analysis, throughout the 3 weeks of culturing, <3% of the proteome was significantly different between FBS and SFM conditions. Only 1 protein, GPX1, was consistently upregulated in spheroids formed in SFM during all 3 weeks of culture. GPX1 is an intracellular antioxidant enzyme that reduces hydrogen peroxide and lipid peroxides and prevents oxidative damage.^54^ As a selenocysteine-containing enzyme, GPX1 expression is regulated by the levels of selenium and selenocysteine incorporation during protein translation.^55^ Here, we hypothesize that additional selenium supplementation from the serum-free cocktail (Supplemental Table 2) during the spheroid formation augments the selenium pool in PHH and therefore increases the expression of GPX1 expression and prevents oxidative stress. At the same time, we observed a consistent upregulation of fatty acid-binding protein 5 in 3D PHH spheroids cultured in FBS. Fatty acid-binding protein 5 has been previously identified as a marker of ferroptosis, a form of programmed cell death characterized by iron-dependent accumulation of reactive oxygen species and diminished antioxidant capacity.^56^^,^^57^

Furthermore, we evaluated the expression and function of the ADMET proteins mentioned in the recently adopted (May 2024) ICH M12 guidelines for drug interactions.^43^ Overall, at a given resolution, the proteins listed in the ICH guidelines were stably expressed in donor 1 in both sets of 3D PHH, and we observed almost no difference between the 2 conditions. We noted some significant expression differences in CYP3A5, as well as in UGT1A3, UGT1A9, and UGT2B4, between 3D PHH formed in FBS-containing medium and those formed in SFM. Besides the significant changes seen, an interesting trend was observed with many of the ADMET proteins showing decreasing expression levels over time in 3D PHH formed in FBS, whereas the corresponding proteins in SFM-formed 3D PHH did not show the same pattern but instead remained stable over time. This was especially evident for CYP2B6, CYP3A4, CYP3A5, and OATP1B3.

Altogether, our data demonstrate that 3D PHH could be formed in 3R-compliant chemically defined medium, without the addition of FBS, and successfully used for in vitro ADMET studies. Morphological and viability evaluations, along with global proteomics data, demonstrated that animal serum-free in vitro liver spheroid cultures are comparable to regular FBS-based in vitro models with regard to the viability and functional performance of P450s. From an ADMET perspective, the use of a defined medium containing only recombinant proteins may be important for improving experimental reproducibility. As mentioned above, the activity of drug transporters in classical model systems such as the Caco-2 cell line is known to be affected by batch-to-batch variability in FBS. Although 3D PHH cultures can be used to study active transport,^20^ they are more commonly applied in drug metabolism studies.^17^, ^18^, ^19^, ^20^, ^21^, ^22^ To our knowledge, no studies have specifically addressed how FBS batch variability might influence drug metabolism in 3D PHH cultures. However, previous research using rat and human primary fetal hepatocytes cultured ex vivo has shown that the concentration of FBS can affect CYP2E1 mRNA and protein expression, as well as enzymatic activity.^58^ Another study using transfected cells demonstrated that the activation of the human constitutive androstane receptor, a nuclear receptor regulating several P450 enzymes, varied depending on the type and source of FBS used.^59^ It should be noted that in standard 3D PHH protocols, FBS is only used during the initial spheroid formation phase and is subsequently diluted out with each medium change. Therefore, any potential influence on P450 metabolism in long-term cultures is likely to be small. Nevertheless, given that we here observed differences in P450 activity, a modest, donor-specific effect cannot be entirely ruled out.

It is important to note that our long-term studies were conducted using only 3 PHH donors, and nonparenchymal liver cells were not included. Therefore, the broader applicability of the SFM to liver cell cultures in general should be interpreted with caution until further validation is available. Although we did not present specific ADMET application data in this study, we believe our findings provide a solid foundation for future context-of-use evaluations. We encourage fellow researchers to consider transitioning ADMET in vitro cultures to serum-free conditions, as this may not only enhance the transparency and reproducibility of experimental outcomes but also support more ethical study designs.

The approach presented in this study and the publications by Rafnsdóttir et al,^8^ Weber et al,^15^ and Oredsson et al^16^ present an opportunity for other researchers for customization of their cell culture medium of choice toward normoglycemic levels of nutrients^17^ and humanized serum-free conditions. Notably, the serum-free substitute is not only applicable for 3D PHH. PHH seeded on collagen-coated cell culture plates using the SFM showed no morphological differences as compared with PHH seeded in FBS-containing medium (Supplemental Fig. 7). In addition, Rafnsdóttir et al^8^ and Weber et al^15^ have successfully used it for culturing a variety of cell lines. Hence, this serum substitute could potentially be used for ADMET cell models. Such projects are currently underway in our laboratory, and preliminary data are encouraging. Of note, there is an opportunity for further improvements of cell culture medium by substitution of human-derived proteins with pure recombinant proteins as mentioned above and/or meticulous evaluation of the role of every component in the cell culture medium.

Lastly, we share our global proteomics data in accordance with the Findable, Accessible, Interoperable, and Reusable data principles, thereby providing a resource for benchmarking the performance of serum-free 3D PHH cultures. These data, together with our experimental findings, may serve as a basis for future studies aimed at developing standardized protocols for 3D PHH cultures, as well as exploring the use of serum-free conditions in primary human cell cultures.

## Conflict of interest

The authors declare no conflicts of interest.
