# Supplementary material for: Animal product–free formation and cultivation of three-dimensional primary hepatocyte spheroids
Source: Drug Metab Dispos. 2025 Aug 19;53(10):100147. doi: 10.1016/j.dmd.2025.100147 (PMC12799525; doi:10.1016/j.dmd.2025.100147)
Supplement: Supplementary Tables 1-9 and Supplementary Figures 1-7 [file mmc1.pdf]

Supplemental information

Animal product-free formation and cultivation of 3D primary hepatocyte spheroids

*Mickols, Evgeniya; Mohammedamin, Rejeen; Primpas, Lazaros; Oredsson, Stina; Karlgren, Maria**

**Address correspondence to: maria.karlgren@uu.se*

Supplemental Table 1. Medical and demographic information of PHH donors used in this study. Age groups: middle-age adult (31-45), old-aged adults (46-75), seniors (>75).

Donor	Sex	Age group	Diagnosis	BMI
1	M	old-aged adult	Colorectal cancer	22
2	F	middle-aged adult	Colorectal cancer	28
3	F	middle-aged adult	Neuroendocrine tumor	23
4	M	senior	Colorectal cancer	29
5	F	senior	Colorectal cancer	20

Supplemental Table 2. The serum-free medium composition.

		William´s E normoglycemic medium		Serum-free supplement	
		**WE P04-29050S4**	**Added in house**	**Rafnsdottir et al.**	**Total**
		mg/L	mg/L	mg/L	mg/L
**Inorganic salts**	NaHCO3	2240			2240
	CaCl2 * 2H2O	264.92			264.92
	$Fe(NO_3)_3$ * 9H2O	0.0001			0.0001
	KCl	400			400
	CuSO4	0.0001			0.0001
	MgSO4	97.67			97.67
	MgCl2 * 4H2O	0.0001			0.0001
	NaCl	6800			6800
	NaH2PO4	140			140
	ZnSO4 * 7H2O	0.0002			0.0002
	Na_2SeO_3/H2SeO3		0.005	0.008	0.013
**Other**	D-glucose		990		990
	Glutathione	0.05		0.012	0.062

	Sodium Pyruvate	25			25
	Dexamethasone		0.392		0.392
	Penicillin		1000 U/mL		1000 U/mL
	Streptomycin		1000 U/mL		1000 U/mL
	All-trans retinoic acid			0.025	0.025
	Cholesterol			0.05	0.05
	Hypoxanthine Na			1.75	1.75
	O-Phosphoryl ethanolamine			5	5
	Pyruvate			110	110
	Ribose			0.125	0.125
	Xanthine			0.085	0.085
	Uracil			0.075	0.075
**Fatty acids**	Linoleic acid			1	1
	Methyllinoleat	0.03			0.03
	Lipoic acid			0.05	0.05
	L-Alanine	90			90
	L-Arginine free base	50			50
	L-Asparagine * H2O	20			20
	L-Aspartic acid	30			30
	L-Cysteine	40			40
	L-Cystine	20			20
	L-Glutamine		292.28		292.28
	L-Glutamic acid	50			50
	Glycine	50			50
	L-Histidine base	15			15

	L-Isoleucine	50			50
	L-Leucine	75			75
	L-Lysine * HCl	87.5			87.5
	L-Methionine	15			15
	L-Phenylalanine	25			25
	L-Proline	30			30
	L-Serine	10			10
	L-Treonine	40			40
	L-Tryptophan	10			10
	L-Tyrosine	35			35
	L-Valine	50			50
**Vitamins**	L-Ascorbic Acid	2		0.012	2012
	D(+)-Biotin	0.5			0.5
	Calciferol/Ergocalciferol	0.1		0.025	0.125
	D-calcium pantothenate	1			1
	Choline chloride	1.5		3.5	5
	Folic acid	1		0.33	1.33
	myo-Inositol	2		4.5	6.5
	Menadione sodium bisulfate	0.01			0.01
	Nicotinamide	1			1
	Pyridoxal * HCl	1			1
	Riboflavin	0.1			0.1
	Thiamine * HCl	1		0.08	1.08
	Alfa-tocopherol phosphate	0.01		0.003	0.013
	Vitamin A acetate	0.1			0.1
	4-Aminobenzoic acid			0.012	

	Vitamin B12	0.2		0.35	0.55
**Hormones**	Insulin		0.00058		0.00058
	Triiodothyronine			0.0000002	0.0000002
	17-beta Estradiol			0.0000005	0.0000005
	Hydrocortisone			0.00025	0.00025
**Proteins and Growth Factors**	Transferrin		5.5	50	55.5
	bFGF			0.001	0.001
	Collagen			0.1	0.1
	EGF			0.01	0.01
	Fetuin			0.04	0.04
	iGF1			0.005	0.005
	Laminin			0.02	0.02
	pDGF			0.002	0.002
	Vitronectin			0.1	0.1
	Human Serum Albumin			1250	1250

Supplemental Table 3. Liquid chromatography gradient events. Mobile phase A1: Water and 0.1% (v/v) formic acid. Mobile phase B1: Acetonitrile and 0.1% (v/v) formic acid.

Time, min	flow rate, µl/min	A1, %	B1, %
Initial	0.5	98	2
0.2	0.5	98	2
1.2	0.5	5	95
1.7	0.5	5	95
1.8	0.5	98	2
2	0.5	98	2

Supplemental Table 4. Metabolite-specific MS parameters.

Compound	Parent ion (m/z)	Product ion (m/z)	Cone voltage (V)	Collision voltage (V)
1-hydroxy midazolam	342.1	203	34	28
1-hydroxy bufuralol	278	158.9	26	22
hydroxy bupropion	256	139	13	27
4-hydroxy diclofenac	312.1	230.1	22	32

Supplemental Table 5. Results of the blinded scoring* of 3D PHH.

Medium	**SFM**	**FBS**	**SFM**	**FBS**	**SFM**	**FBS**	**SFM**	**FBS**	**SFM**	**FBS**
Donor	**D1**	**D1**	**D2**	**D2**	**D3**	**D3**	**D4**	**D4**	**D5**	**D5**
Number when coded	*1*	*9*	*3*	*5*	*8*	*7*	*4*	*6*	*10*	*2*
Researcher 1	3	4	4	3	5	5	5	3	4	4
Researcher 2	4.5	4	4	3	5	4.5	4.5	3	4	4
Researcher 3	4	2	4	2	5	3.5	4	1	4.5	3
Researcher 4	4	3	4	1	5	4	5	2	2	3
Researcher 5	4.5	1.5	3.5	1.5	4	2	3.5	1	2.5	2
**Average**	3.8	2.8	3.8	1.8	4.5	3.6	4.5	2.0	2.8	3.0

***** Excellent 3D PHH morphology (as compactness, clear rim, and no excessive debris) was scored as 5, whereas a score of 1 was indicative of poor compactization and visible cell death.

In Supplemental table 5, one could note that, whilst direction of the rating between SFM and FBS is preserved between researchers, the overall rating differs largely between individual researchers. Thus, we performed inter-rater reliability rating (results in Supplemental table 6).

Supplemental Table 6. Results of interrater agreement with kappa reliability rating.

**Category**	**Kappa**	**SE of kappa**	**95% confidence interval**	**Weighted Kappa**
FBS formed spheroids	-0.211	0.058	-0.325 to -0.096	-0.397
SFM-formed spheroids	-0.100	0.074	-0.244 to 0.044	-0.108

Kappa < 0: No agreement; Kappa between 0.00 and 0.20: Slight agreement; Kappa between 0.21 and 0.40: Fair agreement; Kappa between 0.41 and 0.60: Moderate agreement; Kappa between 0.61 and 0.80: Substantial agreement; Kappa between 0.81 and 1.00: Almost perfect agreement.

Supplemental Table 7. Number of replicates/spheroids included in viability measurements at week 1.

	FBS	***SFM***
Donor 1	20	20
Donor 2	16	16
Donor 3	20	18
Donor 4	20	19
Donor 5	20	20

Supplemental Table 8. Number of replicates/spheroids included in viability measurements during long-term experiments.

	FBS	***SFM***
Donor 1		
Week 1	24	14
Week 2	18	17
Week 3	18	17
Donor 3		
Week 1	15	15
Week 2	15	15
Week 3	15	15
Donor 4		
Week 1	15	15
Week 2	15	15
Week 3	15	15

Supplemental Table 9. Number of replicates/spheroids included in CYP metabolism analyses.

	FBS	***SFM***
Donor 1, week 1		
CYP2B6 (1OH-Bupropion)	24	24
CYP2D6 (1OH-Bufuralol)	24	22
CYP2C9 (4-OH Diclofenac)	24	24
CYP3A4 (1-OH midazolam)	24	17
Donor 1, week 2		
CYP2B6 (1OH-Bupropion)	40	40
CYP2D6 (1OH-Bufuralol)	40	40
CYP2C9 (4-OH Diclofenac)	37	33
CYP3A4 (1-OH midazolam)	39	39
Donor 1, week3		
CYP2B6 (1OH-Bupropion)	36	39
CYP2D6 (1OH-Bufuralol)	33	29
CYP2C9 (4-OH Diclofenac)	33	32
CYP3A4 (1-OH midazolam)	39	39
Donor 3, week 1		
CYP2D6 (1OH-Bufuralol)	12	12
CYP2C9 (4-OH Diclofenac)	12	12
CYP3A4 (1-OH midazolam)	12	12
Donor 3, week 2		
CYP2D6 (1OH-Bufuralol)	12	12
CYP2C9 (4-OH Diclofenac)	12	12
CYP3A4 (1-OH midazolam)	12	12
Donor 3, week 3		
CYP2D6 (1OH-Bufuralol)	12	11
CYP2C9 (4-OH Diclofenac)	12	11
CYP3A4 (1-OH midazolam)	12	12
Donor 4, week 1		
CYP2D6 (1OH-Bufuralol)	12	12
CYP2C9 (4-OH Diclofenac)	12	12
CYP3A4 (1-OH midazolam)	12	12
Donor 4, week 2		
CYP2D6 (1OH-Bufuralol)	12	12
CYP2C9 (4-OH Diclofenac)	12	12
CYP3A4 (1-OH midazolam)	12	12
Donor 4, week 3		
CYP2D6 (1OH-Bufuralol)	12	12
CYP2C9 (4-OH Diclofenac)	12	11
CYP3A4 (1-OH midazolam)	12	12

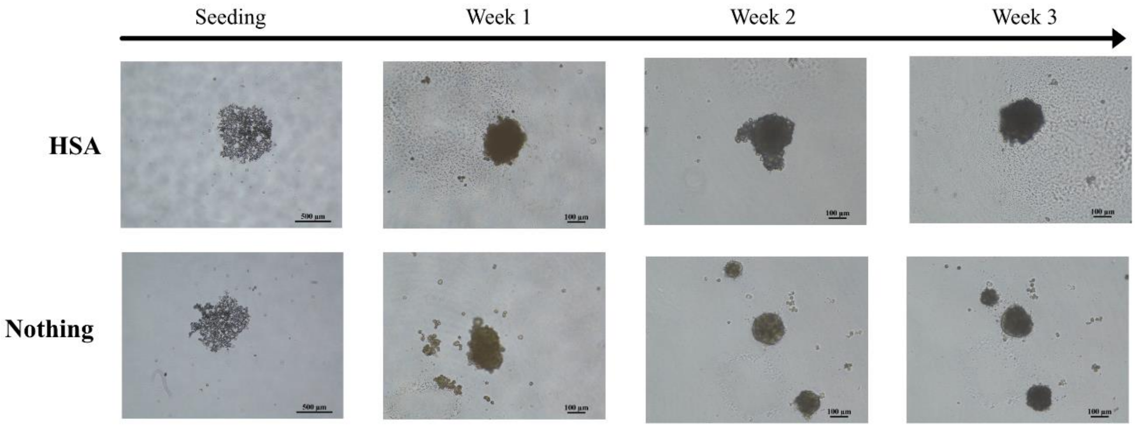

Supplemental Figure 1. PHH formation and culturing for 3 weeks in WEng supplemented with only human serum albumin (HSA) or nothing. Note that the majority of the wells did not form spheroids when cultured without FBS or serum-free supplement. The images shown here represent successful spheroid formation under these conditions which only occurred for 15-20 % of the wells when cultured with HSA or completely without any supplement.

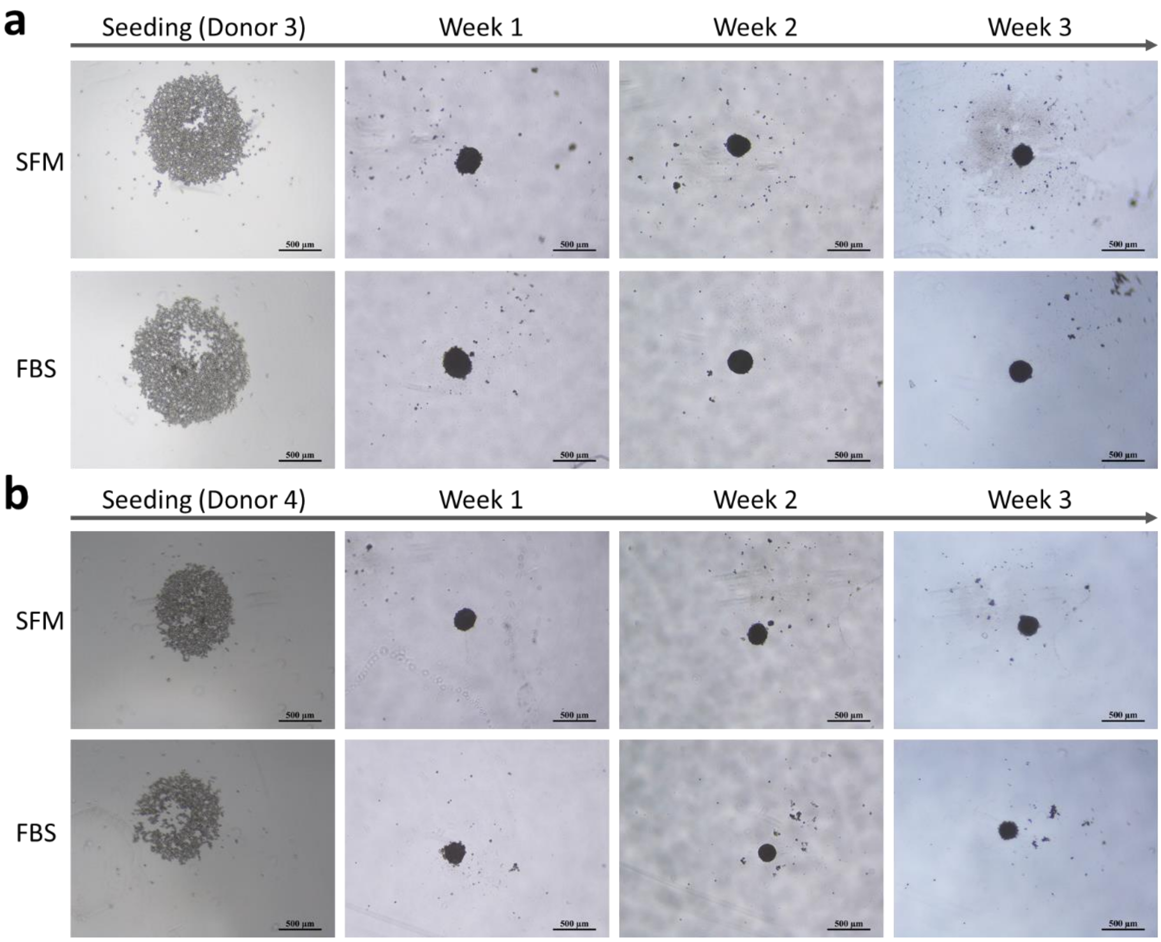

Supplemental Figure 2. 3D PHH spheroid formation in serum-free medium (SFM) or fetal bovine serum (FBS)-supplemented medium and corresponding morphology for three weeks in culture for **a.** Donor 3 and **b.** Donor 4. Scale bar = 500 µm.

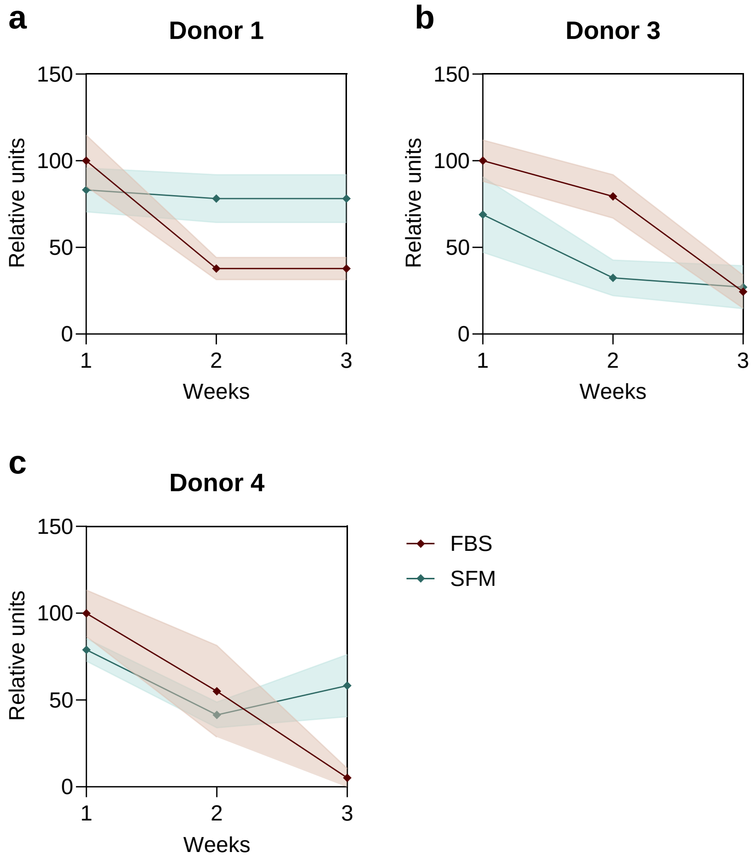

Supplemental Figure 3. Viability (ATP content) measured over three weeks in culture in serum-free medium (SFM; green) or fetal bovine serum-supplemented medium (FBS; brown) for **a.** Donor 1, **b.** Donor 3, and **c.** Donor 4. The shaded area represents standard deviation (n=15-24, see Supplemental Table 8 for detailed information).

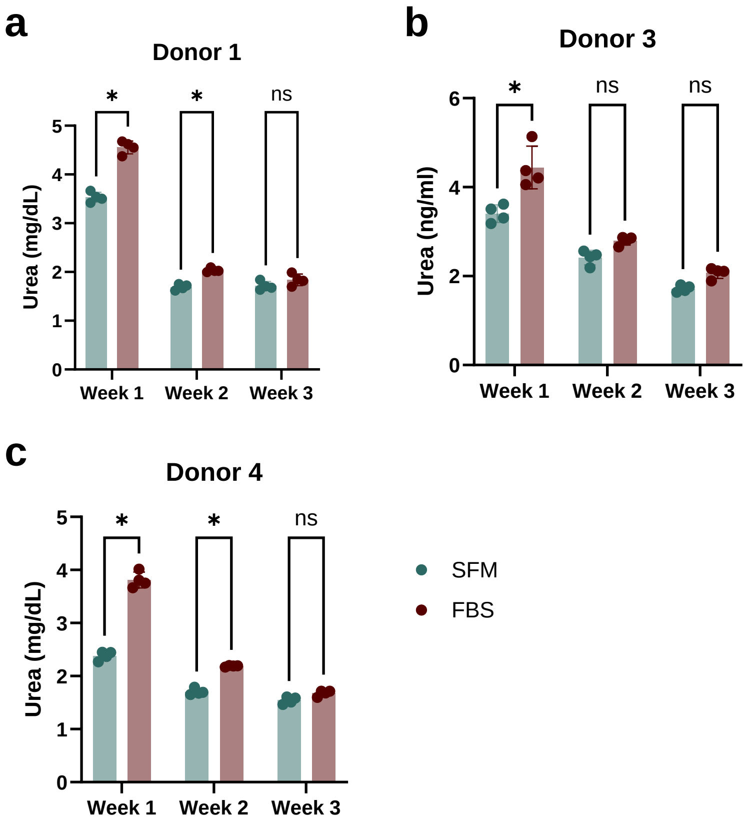

Supplemental Figure 4. Urea production in 3D PHH for three weeks in culture in serum-free medium (SFM; green) or fetal bovine serum-supplemented medium (FBS; brown) for **a.** Donor 1, **b.** Donor 3, and **c.** Donor 4. Data are presented as mean ± standard deviation (n=4, where each replicate consists of medium pooled from six spheroids). *, $p < 0.0001$; ns, not significant using two-way ANOVA with Šídák's multiple comparisons test.

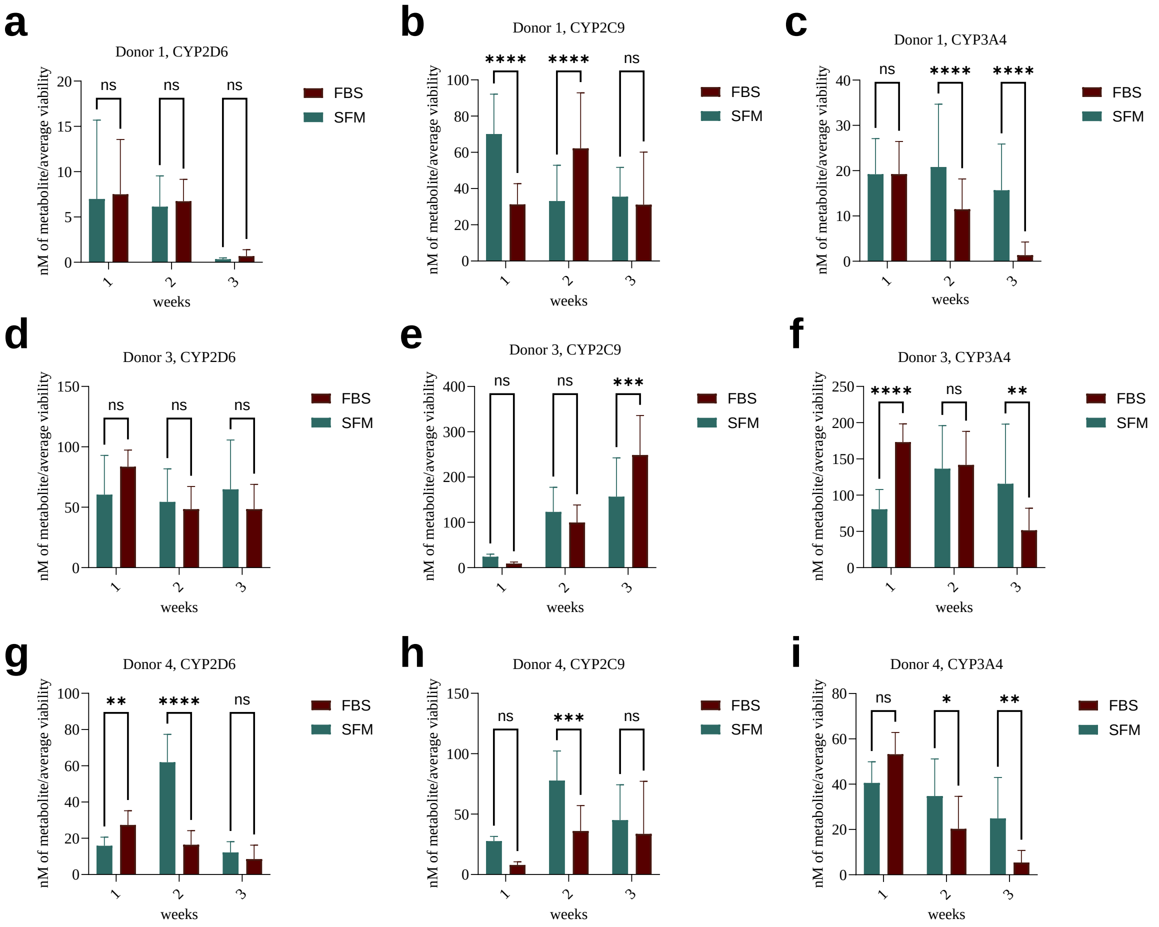

Supplemental Figure 5. Marker metabolite formation in nM normalized to average viability in spheroids cultured for one, two, and three weeks. Data are presented as mean ± standard deviation (n=11-40, see Supplemental Table 9 for detailed information). *, $p < 0.05$; **, $p < 0.01$; ***, $p < 0.001$ ****, $p < 0.0001$; ns, not significant using two-way ANOVA with Šídák's multiple comparisons test.

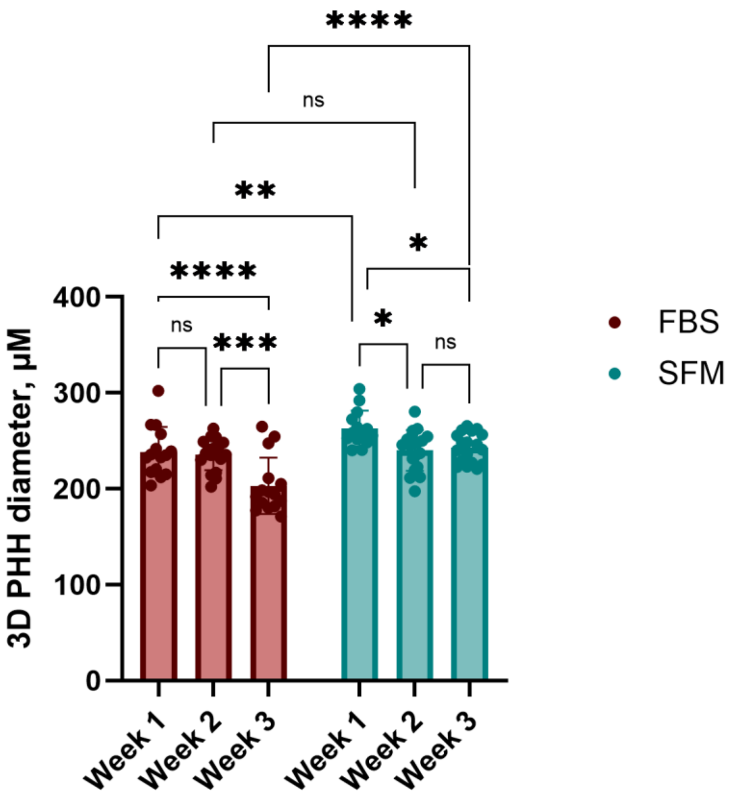

Supplemental Figure 6. Donor 1 3D PHH diameter at every week of culturing. Overall, a certain degree of compactization occurs in both conditions. One could observe a slight decrease in diameter by the third week of culturing in spheroids formed in fetal bovine serum (FBS)-containing medium, while in serum-free medium (SFM) formed spheroids this trend is less visible. Statistical significance evaluated in two-way ANOVA with Šídák's multiple comparisons test. n=15-19.

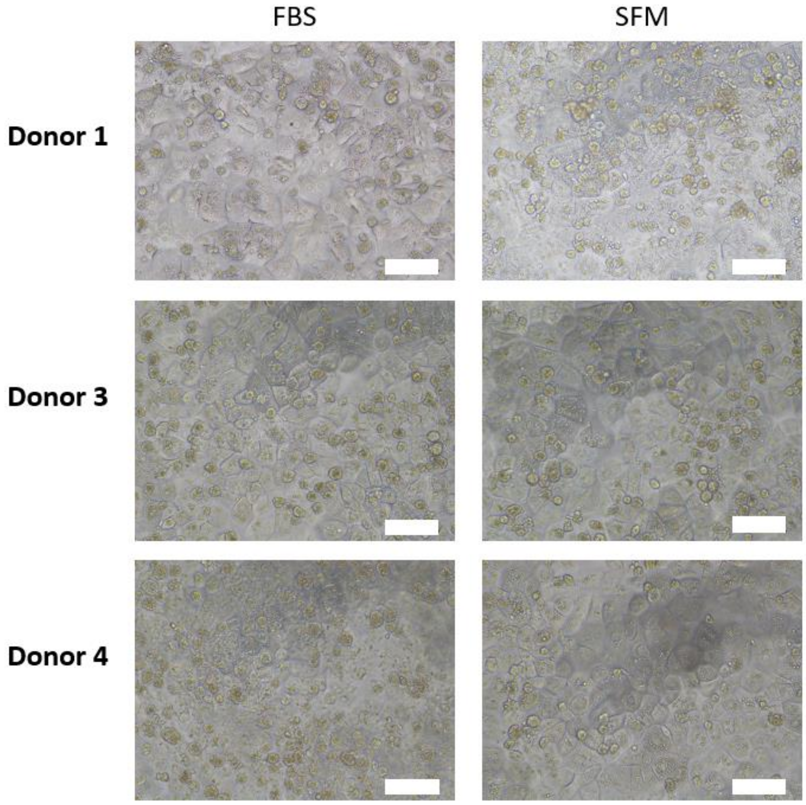

Supplemental Figure 7. Phase-contrast microscopy images showing the 2D monolayer morphology of primary human hepatocytes derived from three individual donors (Donor 1, Donor 3, and Donor 4). Cells were seeded on collagen-coated 24-well plates (300 000 cells/well) and cultured in fetal bovine serum (FBS)-containing medium or in serum-free medium (SFM). Images were captured after 24 hours of culture. Hepatocytes cultured in both FBS and SFM conditions showed characteristic polygonal morphology and cell-cell contact, though donor-specific differences in cell density and morphology can be seen. Scale bar = 100 µm.
